# Acute leukemia in adult Hispanic Americans: a large-population study

**DOI:** 10.1038/bcj.2016.94

**Published:** 2016-10-14

**Authors:** R Swords, J Sznol, R Elias, J Watts, A Zelent, E Martin, F Vargas, S Bethel-Ellison, E Kobetz

**Affiliations:** 1 Leukemia Program, Sylvester Comprehensive Cancer Center, Miller School of Medicine, University of Miami, Miami, FL, USA; 2 Jay Weiss Institute for Public Health Sciences, Sylvester Comprehensive Cancer Center, Miller School of Medicine, University of Miami, Miami, FL, USA; 3 Center for Genetic Epidemiology and Statistical Genetics, John P. Hussman Institute for Human Genomics, Miller School of Medicine, University of Miami, Miami, FL, USA; 4 Internal Medicine Residency Program, University of Miami/JFK Medical Center Palm Beach Regional GME Consortium, Miami, FL, USA

Acute leukemia (AL) is a diverse group of clonal hematopoietic disorders that are broadly categorized into two types: acute myeloid leukemia (AML) and acute lymphoblastic leukemia (ALL).^1, 2, 3^ AML is further grouped into acute promyelocytic leukemia (APL, a highly curable disease with a unique and pathognomonic genetic lesion) and non-APL AML. ALL is also further lineage classified into B-cell ALL or T-cell ALL. Unlike older classification systems that defined AL according to how leukemia cells looked and stained under the light microscope,^1^ the current iteration of the World Health Organization (WHO) classification of hematopoietic neoplasms incorporates cytogenetic and molecular data to provide prognostic and therapeutic information of value to the treating clinician.^2, 3^ When applied to large-population databases, the WHO framework also provides useful insights into the distribution of AL among ethnic groups, informing on the causative factors of AL, which are at this time poorly understood. For example, in the United States, whites have higher rates of AML compared with other groups.^4^ B-ALL is uncommon in blacks and Asians, whereas Hispanics have the highest incidence rates (IR) of this leukemia.^5^ AML appears less common in Hispanics when compared with whites, however, APL appears comparatively more common in Hispanics.^6^ The varied distribution of AL among these ethnic groups suggests that host susceptibility factors are critical determinants of disease in one group, but not in another. The extent to which the environment interacts with these factors is unknown. In Florida, Hispanics comprise 23.6% of the population, with up to 65% of this group residing in South Florida. About 51% of Hispanics in Florida are native born, 49% are foreign born. In 2015, it is estimated that 3930 new cases of AL will be diagnosed.^7^ Given the known interaction between ethnicity and AL incidence, we sought for the first time, to better understand the epidemiological patterns of AL distribution throughout Florida. Utilizing the Florida Cancer Data System (FCDS), we analyzed the patterns of B-cell ALL, T-cell ALL, non-APL AML and APL AML among Hispanics and non Hispanic Whites.

Cancer incidence data were obtained from the FCDS from 2004 to 2013. The FCDS is a cancer registry for the entire state of Florida, and a member of the National Program of Cancer Registries administered the Centers for Disease Control and Prevention (CDC). FCDS data collection practices are described elsewhere.^7^ Inclusion criteria for this study included all patients over the age of 20, Florida residents, identified as White or Hispanic of any race, and diagnosed with of B-cell ALL, T-cell ALL, non-APL AML and APL AML, utilizing the International Classification of Diseases for Oncology 3rd Edition histology codes. B-Cell ALL is defined as histologic codes 9727, 9728, 9835, 9836, T-cell ALL is defined as 9729, 9837, non-APL AML is defined as 9840, 9861, 9867, 9870, 9871,9872, 9873, 9874, 9891, 9895, 9896, 9897, 9910, 9920, 9930, 9931 and APL is defined as 9866. These four leukemias were chosen for analysis given previous research on the association of race and ethnicity with each and the differing clinical aspects of each. Population estimates, separated by race/ethnicity, were obtained from the 2010 SF1 100% census data. Age-adjusted IR and incidence rate ratios (IRR), per 100 000, were adjusted to the 2000 standard US million population. The 95% confidence intervals were calculated for IR and IRR using the normal approximation. Age was categorized in 15-year age groups.

Table 1 summarizes the IR as well as the IRR of ALL (both B- and T-cell ALL) and AML (APL and non-APL cases) in both non Hispanic whites and Hispanics. Similarly in Table 1, IR and IRRs are presented for these AL subtypes for both US-born and non-US-born Hispanics. Our results agree with previous reports, where Hispanics have higher rates of B-ALL compared with non Hispanic whites. Hispanics also had comparatively higher rates of APL and unlike previous reports; AML, in general, was more common in Hispanics (regardless of subtype). Also in agreement with other reports, T-cell ALL was less common in Hispanic patients. Our nativity analysis confirmed no significant difference in AL distribution between US-born and foreign-born Hispanics. Finally, to assess the effects of age on these findings, the age distribution of AL by 15-year increments is presented in Table 2. Because prognosis and treatment outcomes differ significantly between adult and pediatric patients, and because nativity analyses including children have the potential for age-related bias, we restricted our study to an adult population. The age distribution trends in Table 2 reflect what is typically known about the IR of AL in adult patients.

The lack of population-based studies in AL relate to a large degree on the rarity of these diseases when compared with other tumor types. Nonetheless, when compared with similar reports analyzing the population distribution of AL in large populations.^8^ Our findings are in general agreement. We noticed, however, that AML was generally more common in Hispanics compared with non Hispanic whites, regardless of subtype. Typically, other studies have reported that APL is more common in Hispanics but that the incidence of AML is less common overall, when compared with whites.^6, 8^ In a California analysis, it was noted that 84% of Hispanics in that state are of Mexican origin.^8^ In our analysis, Hispanics of Mexican descent were a minority group, which may explain, in part, this new finding. In agreement with others,^8, 9^ lack of a nativity difference implies that host susceptibility factors are critical determinants of disease pathogenesis in the case of AL. For example, Douer *et al.*^10^ showed that a specific break-point variant (bcr-1) of the driver chimeric fusion gene in APL was more common in Hispanics than non-Hispanics. Similarly, single-nucleotide polymorphisms in the *GATA3* gene appear more common in Hispanics with B-ALL compared with other groups.^11^ Several other so-called ‘risk alleles' including ARID5B, CDKN2A and CEBPE have also been found more commonly in Hispanic B-ALL.^12^ The interaction between environmental factors and these host susceptibility factors in the pathogenesis of AL is very much, unchartered territory. Several environmental modifiers have been implicated and include infectious agents (viruses mainly), ionizing radiation, herbicides, embalming fluids, ethylene oxides and smoking.^13, 14^ As we observed no effect from nativity in our analysis, it is difficult to implicate specific environmental factors that might contribute directly to the onset of AL in this adult population. We acknowledge, of course, that the age at immigration could impact on non-American environmental exposures however; this data were not available in our analysis.

AL is a medical emergency, early diagnosis and treatment heavily influence outcome. In Hispanic children with B-ALL, poorer socioeconomic status clearly influences overall survival.^14^ There are limited data relating socioeconomic status to survival in adults with AL. Overall, almost a quarter of Hispanics live in poverty in the United States.^15^ Most of these will be uninsured. Older Hispanic patients, particularly, are less likely to have health insurance when compared with non Hispanic white patients (44.6% vs 25.7%, respectively). In South Florida, 33% of Hispanics are uninsured and a significant poverty disparity exits (19% vs 12% for non Hispanic whites). These factors can contribute to poorer outcome in Hispanic populations.

In conclusion, we report a higher incidence rate of B-ALL for Hispanics in Florida. In addition, we also noted that AML (regardless of subtype) was more common in Floridian Hispanics. The absence of a nativity difference in accounting for variation in IR implies that heritable factors are key determinants of disease pathogenesis.

## Figures and Tables

**Table 1:** Age-adjusted incidence rates of leukemia subtypes by race/ethnicity

*Acute leukemia subtype*	*Race/ethnicity or nativity*	*Cases*	*Crude incidence per 100 000*	*Age-adjusted incidence per 100 000*	*Incidence rate ratio (95% CI)*
B cell	Non Hispanic White	794	1.260	0.120	1 (Reference)
	Hispanic	306	0.485	0.195	1.627* (1.426–4.161)
T cell	Non Hispanic White	65	0.103	0.105	1 (Reference)
	Hispanic	19	0.030	0.162	1.548 (0.928–2.530)
Non-APL	Non Hispanic White	6652	10.554	0.074	1 (Reference)
	Hispanic	1076	1.707	0.113	1.533* (1.437–4.208)
APL	Non Hispanic White	464	0.736	0.052	1 (Reference)
	Hispanic	146	0.232	0.068	1.302* (1.081–2.947)
B cell	US born	456	0.723		1 (Reference)
	Foreign born	260	0.413		0.570 (0.490–1.632)
T cell	US born	53	0.084		1 (Reference)
	Foreign born	21	0.033		0.396 (0.239–1.270)
Non-APL	US born	2401	3.809		1 (Reference)
	Foreign born	977	1.550		0.407 (0.378–1.459)
APL	US born	222	0.352		1 (Reference)
	Foreign born	89	0.141		0.401 (0.314–1.368)

Abbreviations: APL, acute promyelocytic leukemia; CI, confidence interval.

Age-adjusted incidence rates (per 100 000) of adult acute leukemias, incidence rate ratios by race/ethnicity (2004–2013) and nativity among Hispanics in Florida. * Statistically significant.

**Table 2:** Age-adjusted incidence rates of leukemia subtypes by race/ethnicity and 15-year age group

*Acute leukemia subtype*	*Age range*	*Race/ethnicity*	*Cases*	*Crude incidence per 100 000*	*Age-adjusted incidence per 100 000*	*Incidence rate ratio (95% CI)*
B cell	20–34	Non Hispanic white	109	0.668	0.189	1.00
		Hispanic	85	1.302	0.369	1.95*(1.47–4.34)
	35–49	Non Hispanic white	147	1.026	0.338	1.00
		Hispanic	78	1.605	0.528	1.56* (1.20–3.31)
	50–64	Non Hispanic white	212	1.095	0.230	1.00
		Hispanic	82	2.189	0.460	2*(1.55–4.71)
	65–79	Non Hispanic white	221	2.295	0.299	1.00
		Hispanic	47	3.338	0.435	1.45* (1.06–2.89)
	80+	Non Hispanic white	105	3.102	0.145	1.00
		Hispanic	14	3.458	0.162	1.11 (0.64–1.89)
T cell	20–34	Non Hispanic white	20	0.123	0.035	1.00
		Hispanic	8	0.123	0.035	1 (0.44–1.55)
	35–49	Non Hispanic white	13	0.091	0.030	1.00
		Hispanic	6	0.123	0.041	1.36 (0.56–1.76)
	50–64	Non Hispanic white	11	0.057	0.012	1.00
		Hispanic	1	0.027	0.006	0.47 (0.06–1.06)
	65–79	Non Hispanic white	13	0.135	0.018	1.00
		Hispanic	4	0.284	0.037	2.1 (0.69–1.99)
	80+	Non Hispanic white	8	0.236	0.011	
		Hispanic	0	0.000	0.000	
Non-APL	20–34	Non Hispanic white	207	1.268	0.359	1.00
		Hispanic	96	1.470	0.417	1.16 (0.91–2.48)
	35–49	Non Hispanic white	490	3.421	1.057	1.00
		Hispanic	163	3.354	1.104	1.04 (0.84–2.31)
	50–64	Non Hispanic white	1441	7.441	1.565	1.00
		Hispanic	226	6.033	1.269	0.81 (0.70–2.02)
	65–79	Non Hispanic white	2705	28.086	3.664	1.00
		Hispanic	392	27.840	3.632	0.99 (0.89–2.44)
	80+	Non Hispanic white	1809	53.439	2.499	1.00
		Hispanic	199	49.147	2.298	0.92 (0.79–2.21)
APL	20–34	Non Hispanic White	41	0.251	0.071	1.00
		Hispanic White	21	0.322	0.091	1.28 (0.76–2.13)
	35–49	Non Hispanic White	90	0.628	0.207	1.00
		Hispanic White	51	1.049	0.345	1.67* (1.04–2.83)
	50–64	Non Hispanic White	127	0.656	0.138	1.00
		Hispanic White	36	0.961	0.202	1.47* (1.01–2.75)
	65–79	Non Hispanic White	163	1.692	0.221	1.00
		Hispanic White	28	1.989	0.259	1.17 (0.79–2.20)
	80+	Non Hispanic White	43	1.270	0.059	1.00
		Hispanic White	10	2.470	0.116	1.94 (0.98–2.66)

Abbreviations: APL, acute promyelocytic leukemia; CI, confidence interval.

Age-adjusted incidence rates (per 100 000 person years) of adult acute leukemias and incidence rate ratios by race/ethnicity (2004–2013) among whites and Hispanics, as well as nativity data for Hispanics. All data are stratified by 15-year age groupings. * Statistically significant.
